# Alloplastic Reconstruction of the Extensor Mechanism after Resection of Tibial Sarcoma

**DOI:** 10.1155/2011/545104

**Published:** 2011-04-11

**Authors:** Boris Michael Holzapfel, Hans Rechl, Stefan Lehner, Hakan Pilge, Hans Gollwitzer, Erwin Steinhauser

**Affiliations:** ^1^Orthopaedic Center for Musculoskeletal Research, Department of Orthopaedic Surgery, König-Ludwig-Haus, Julius-Maximilians-University Würzburg, Brettreichstraße 11, 97074 Würzburg, Germany; ^2^Clinic for Orthopaedic Surgery and Traumatology, Klinikum Rechts der Isar, Technical University Munich, Ismaninger Str. 22, 81675 Munich, Germany; ^3^Department of Precision- and Micro-Engineering, Engineering Physics, Munich University of Applied Sciences, Lothstraße 34, 80335 Munich, Germany

## Abstract

Reconstruction of the extensor mechanism is essential for good extremity function after endoprosthetic knee replacement following tumor resection. Only a few biological methods have been able to reliably restore a functional extensor mechanism, but they are often associated with significant complication rates. Reattachment of the patellar tendon to the prosthesis using an alloplastic patellar ligament (Trevira cord) can be an appropriate alternative. In vivo and in vitro studies have already shown that complete fibrous ingrowth in polyethylene chords can be seen after a period of six months. However, until now, no biomechanical study has shown the efficacy of an alloplastic cord and its fixation device in providing sufficient stability and endurance in daily life-activity until newly formed scar tissue can take over this function. In a special test bench developed for this study, different loading regimes were applied to simulate loads during everyday life. Failure loads and failure modes were evaluated. The properties of the cord were compared before and after physiological conditioning. It was shown that rubbing was the mode of failure under dynamic loading. Tensile forces up to 2558 N did not result in material failure. Thus, using an artificial cord together with this fixation device, temporary sufficient stable fixation can be expected.

## 1. Introduction

Until the early 1970s, amputation was the only alternative in the treatment of malignant musculoskeletal tumors. But, even then the five-year survival rates of patients with osteosarcoma reached only 20 percent. However, since the introduction of chemotherapy and radiotherapy, survival rates have increased significantly, that is, up to 70 percent in osteosarcoma. Adjuvant chemotherapy in combination with local resection can keep the local recurrence rate below 5% [1, 2]. By using special therapeutic regimen, the incidence of metastatic disease in limb salvage is similar to that using amputation alone [3]. Therefore, the number of patients eligible for limb salvage procedures after wide resection of tumors has been increased continuously [4]. For reconstruction of skeletal defects after local tumor resection, both biological techniques (e.g., autografts, allografts, rotation plasty) and megaprostheses are used. These procedures, mainly allograft reconstruction and endoprostheses, are well established and have been well described in the literature [5–8]. Depending on the extent and localization of the tumor, soft tissue loss within a wide tumor resection yields several problems. Especially in tumors of the proximal tibia and the knee joint, reconstruction of the extensor mechanism can be a challenging problem [9, 10]. The integrity of the extensor system is essential for good extremity function, and good soft tissue coverage may be difficult to achieve [11–13]. Different reconstructive techniques are described to restore the extensor mechanism and to avoid an extension lag: direct reattachment of the patellar tendon to the prosthesis [14], pedicled muscle transpositions [13, 15–17], reinforcement with an autologous bone graft [18, 19], and allograft reconstruction [20, 21]. Only a few biological methods, however, have been able to reliably restore a functional extensor mechanism [22]. 

Reattachment of the patellar tendon to the prosthesis with synthetic material can be a promising alternative. In previously released reports, the alloplastic cord was sutured to the remainder of the patellar ligament on one end while the opposite end was fixed to the tibial implant by clamps, staples, or blocks [23–25]. In 2002, a clinical report on the latter reconstruction mode was given by our working group whereat the results were encouraging [25]. Textile implants were first introduced for reconstruction of the extensor mechanism in failure of conventional total knee arthroplasty [26, 27] and reconstruction of the cruciate ligaments [28, 29]. Since then, more and more possible indications were added [30–33]. Therefore, these implants have been extensively tested both experimentally and clinically [34–37]. 

In vivo and in vitro studies have already shown that complete fibrous ingrowth in polyethylene chords can be seen after a period of six months [38]. From then on, newly formed scar tissue can assume extensor function, while the capacity of an alloplastic cord in providing durable stability seems to be limited [23]. Clinical studies corroborate this belief [24, 25, 39]. But until now, no biomechanical study has verified the efficacy of an alloplastic cord and its fixation device in providing stability and good functional results in patients' everyday life after reconstruction of the extensor mechanism following proximal tibial replacement. The aim of this study was to test the endurance of this particular cord (Trevira cord) and its fixation device during normal daily life activities, like level walking, stair climbing, or rising from a chair. 

The mechanical properties of an alloplastic cord are shown to change under functional loading and under in vivo conditions. Therefore, we compared the quasistatic properties of the Trevira cord before and after physiological conditioning. Changes in quasi-static failure load and failure mode following low-cycle fatigue were evaluated. Furthermore, the number of cycles to failure and failure mode for various simulated angle combinations and for two-edge designs of the fixation block was determined. 

## 2. Materials and Methods

**Figure 1:**
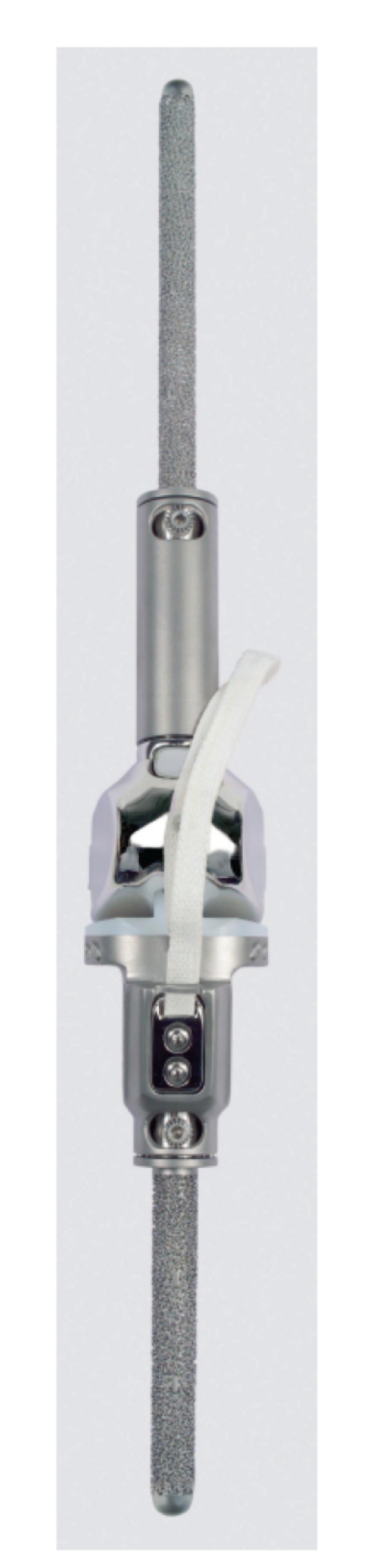
MML tumor knee for proximal tibial and distal femoral replacement (Modular endoprosthetic system Munich-Luebeck, Eska Orthodynamics GmbH, Luebeck, Germany) with the tibial fixation device for the double-layered Trevira cord.

In an experimental setup, the proximal tibial component of the modular MML tumor prosthetic system (Modular endoprosthetic system Munich-Luebeck, Eska Orthodynamics GmbH, Luebeck, Germany) was tested (Figure 1). All metal parts of this system are made of CoCrMo alloy. It has been in clinical use since 1994. The modular construction allows substituting or bridging any kind of bone defect. The most frequent indications are tumors followed by revision arthroplasty due to loosening, periprosthetic fractures, and joint resection surgery due to infection [40, 41].

Biomechanical tests were carried out with a 10 mm wide and 1 mm thick Trevira cord (Telos, Hungen-Obbornhofen, Germany) as it is in clinical use [25]. The material complies with the requirement § 177.1630, polyethylene phthalate polymers, of the Code of Federal Regulations, Food and Drug Administration (FDA/USA) from 4 January 1979. In vivo, the alloplastic cord loops the patella and it is proximally fixed at the remaining tissue of the extensor mechanism by nonresorbable sutures. Our test setup simulated exactly the fixation device as it is in clinical use. Both cord ends were grasped one on top of each other in a metal block which was fixed to the tibial component with two screws penetrating block and cord. Two different fixation block designs were tested. One was the original block as currently in clinical use and the other one a modification of it, comprising an optimized smoothened surface at the proximal edge where the ligament exits the block with oblong holes for screw fixation instead of the regular drill holes. 

Due to the fact that every fixation method of a ligament causes marked shortcomings in tensile strength compared to the ultimate tensile strength of the cord itself, we performed the following test procedures.

1. Quasi-static tensile loading to rupture (test velocity 5 mm/min), pulling parallel to tibial axis. Ligament specimens were tested as received and after conditioning in saline at 37°C over 25 days (each group *n* = 5).
2. Quasi-static loading to 500 N, then 100 cycles of loading between 200 and 500 N at 1 Hz, final quasi-static loading until rupture (test velocity 5 mm/min), pulling parallel to tibial axis (*n* = 10).
3. Cyclic loading under willful neglect of the varying angle between cord and tibia, pulling parallel to tibial axis (frequency = 2.6 Hz; *n* = 7).
4. Cyclic loading at different maximum load levels at anatomically relevant angles with a frequency of 2.6 Hz. With the regular fixation block, the load regimes for stair climbing (*n* = 9) and walking (*n* = 9) were tested. With the modified block, the load regimes for walking (*n* = 10) and rising from a chair (*n* = 2) were investigated in the same manner.

For the tests described under (1)–(3), only the original fixation block was used. 

**Figure 2:**
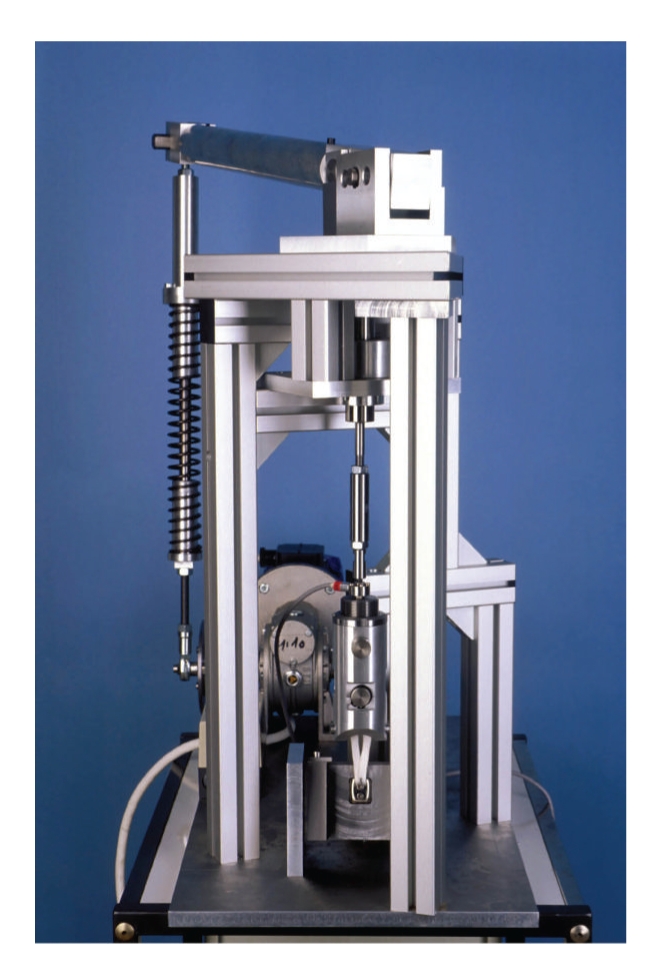
Test bench for cyclic load application on artificial ligament and fixation block.

The test setup is shown in Figure 2. Quasi-static tests were performed on a universal testing machine (Wolpert TZZ 707/50 kN, Ludwigshafen, Germany). During the test, load and displacement were recorded. For the dynamic tests without variation of the ligament angle, a servohydraulic testing machine (Roell Amsler REL 2151/20 kN, Gottmadingen, Germany) was used under load control. Load and displacement were recorded as well. The parameters tensile load to rupture and ligament stiffness before and after conditioning in saline at 37°C over 25 days were subjected to statistical analysis (MAPLE 11, Student's *t*-test). Mean values and standard deviations were calculated, a nominal alpha level of .05 was preselected for the assessment of differences by conditioning.

To simulate the varying angle between cord and tibia during activities, like level walking, stair climbing, or rising from a chair, a special test bench was developed (Figure 2). Thus, the stress could be applied to the artificial patellar ligament at varying degrees of knee flexion and ligament angle of emergence, that is, the angle between the patellar ligament and tibial axis. The different activities were simulated by changing the synchronization between these parameters. The necessary data were obtained from the literature.

Hirokawa described the quadriceps force as a function of the knee flexion angle for different activities like walking or stair climbing [42]. Ellis et al. evaluated knee joint forces while rising from a chair in an experimental, study [43]. In a three-dimensional mathematical model Hirokawa introduced the ratio of patellar ligament force to quadriceps force as a function of knee flexion in a normal knee [44]. At full extension the ratio is approximately equal and changes with increasing flexion angle to 0.75 for a flexion angle of 120°. A decreasing ratio for an increasing flexion angle of the knee joint was also ascertained by Ellis et al. [45], who reported that the force ratio varied from 1.0 to 0.5 under dynamic loading conditions and by Huberti et al. [46], who reported a range of 1.25 to 0.75 under static loading conditions. Van Eijden et al. [47] showed numerically that this ratio decreases from 1.1 at maximal extension to 0.55 at 80° flexion. Sharma et al. [48, 49] analyzed the ratio after the implantation of a knee endoprosthesis. Van Eijden et al. [50] described an almost linear relationship between knee flexion and the ligament angle of emergence. Under physiologic conditions, the ligament undergoes a backward rotation of approximately 35° relative to the tibia during flexion of 120°. From the tibial tuberosity, the patellar ligament points anteriorly between 0° and 80° of flexion and posteriorly between 80° and 120° of flexion. During a gait cycle, the knee flexes to 60°. During stair climbing, the flexion angle changes between 10° and 90° and during rising from a chair about 100° [42, 43, 51–54].

**Figure 3:**
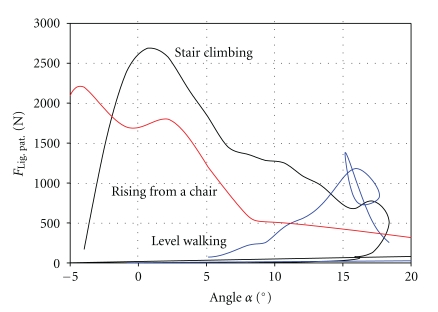
Load regimes for different activities.

By combining the literature data cited above, a peak load for level walking of 1400 N was calculated in the patellar ligament when the patellar ligament points 15° anteriorly compared to the tibial axis. For stair climbing, the peak load was determined to be 2700 N when the patellar ligament is pulling approximately parallel to the tibial axis. Rising from a chair results in a load of 2200 N when the patellar ligament points 4° posteriorly (Figure 3). 

## 3. Results

**Figure 4:**
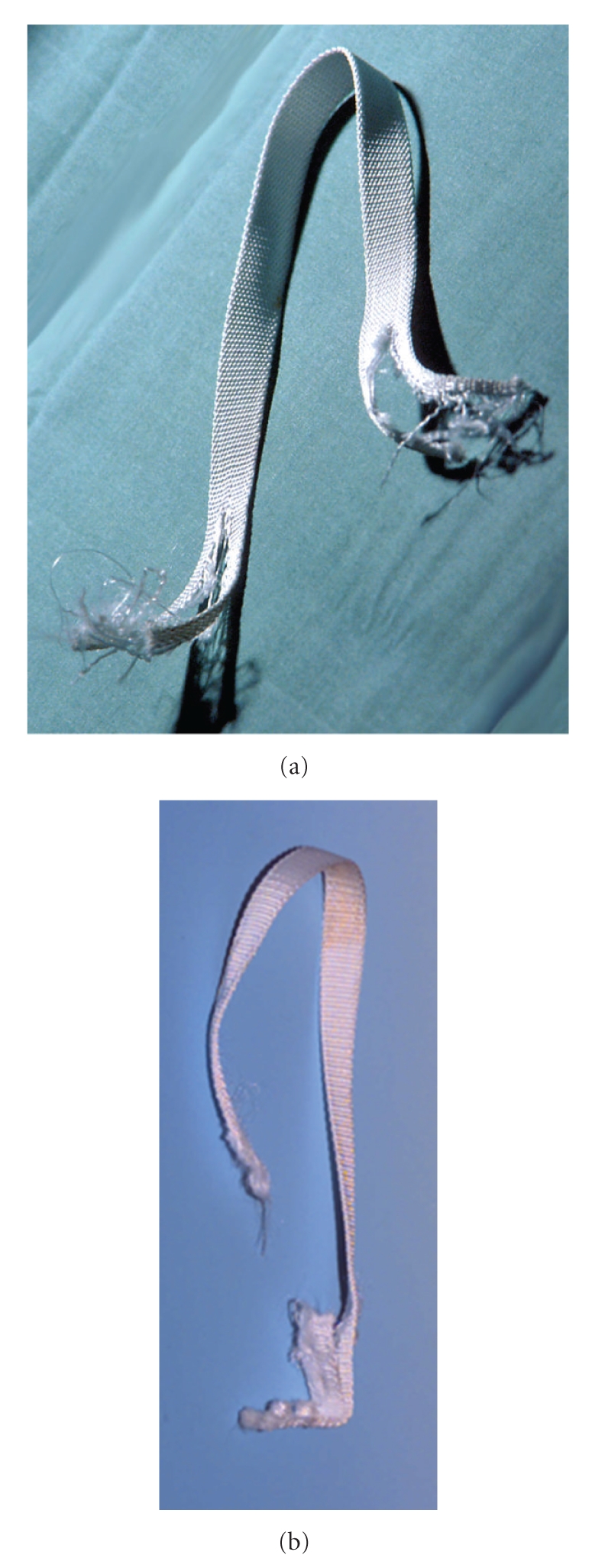
Ruptured Trevira cord after quasistatic loading (a) and (b) after cyclic loading.

**Figure 5:**
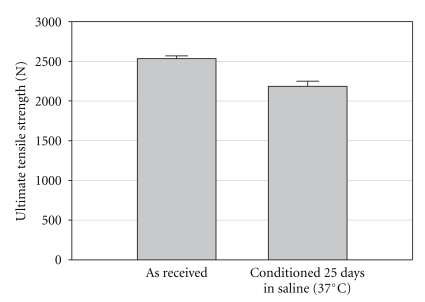
Ultimate tensile strength of the artificial ligament fixed in the block.

The cord ruptured at a mean quasi-static tensile load of 2558 N (SD = 40 N) at the proximal fixation hole of the metal block (Table 1). Initial cracks at this critical cross-section of the cord lead to a complete rupture (Figure 4(a)). The load to failure was statistically significantly reduced by approximately 12% to 2248 N (SD = 88 N) when Trevira cords were kept for 25 days in saline at 37°C before testing (*P* = .007) (Figure 5). The stiffness before plastic deformation of the fixation mechanism was reduced from 136.5 N/mm SD = 4.9 N/mm) to 126.0 N/mm (SD = 9.1 N/mm) after conditioning in saline (Table 1). This 8% decrease was statistically not significant (*P* = .14).

Table 2 shows the ultimate quasi-static tensile strength after one quasi-static load cycle up to 500 N and after 100 cycles of dynamic loading between 200 and 500 N. Compared to the quasi-statically loaded cords, the ultimate load after this low-cycle fatigue test decreased from 2558 N to 2015 N. Failure always occurred at the proximal screw fixation hole.

**Figure 6:**
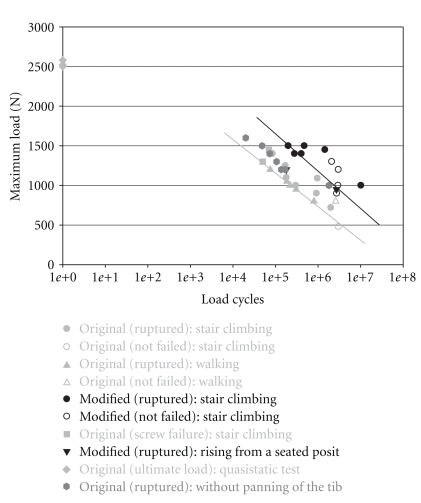
The Woehler curve showing the load cycles to failure for the two different fixation blocks (originally and modified) and different loading regimes.

Under dynamic conditions, the cord scrubbed at the proximal edge of the metal block until it ripped at this point (Figure 4(b)). In terms of the number of cycles to failure, the Woehler curve (Figure 6) showed a significant difference between a block with a modified surface finish at the proximal edge and the original block. Improved edge design led to higher endurance of the artificial ligament as shown by the times-to-failure curve shifting to higher cycles. 

No significant influence was observed regarding the ratio of maximum load and corresponding ligament angles of emergence. Tests performed with the original block in the servohydraulic testing machine (without swiveling of the tibial component) showed the same load-dependent lifespan as tests with varying ligament angles performed in the test bench. The results determined with the modified block did not show any difference between load regimes of the level walking either rising from a chair (Figure 6).

In two cases in which the original block did not perfectly fit its notch, the fixation screws fractured due to dynamic bending. In tests using the block with oblong screw holes no failures occurred due to the absence of a gap between the block and the proximal edge of its notch thus avoiding bending and shear to the screws. 

## 4. Discussion

This study sought to evaluate the quasi-static and dynamic properties of the Trevira cord for reconstruction of extensor mechanism after tibial replacement. Several mechanical test regimes were explored to determine the effect of physiological conditioning, cyclic loading at various load levels, the effect of optimized fixation, and the incorporation of more physiological kinematics in the mechanical loading systems. 

In previous works, the Trevira cord has been analyzed for artificial replacement of the anterior cruciate ligament and different statements about properties of artificial cords in vivo are documented. Siebert et al. [55] stated that the mechanical properties of artificial cords clearly deteriorate under in vivo conditions. The ultimate load of a Trevira cord after 500 days of implantation in a sheep decreased approximately 17%, and the elongation under loading of 500 N increased 2.4% compared to a cord as received. On the other hand, Letsch [37] and Contzen [56] found that this artificial cord is an ideal solution for the biomechanical and biological requirements of cruciate ligament prostheses. Contzen found in his experimental study that the ultimate load of a Trevira cord after 170 days of implantation in the stifle joint of a sheep decreased by approximately 10%. Elongation under physiological relevant loading of 500 N increased from 1.8% to 2.5% in the same period. In our investigation, the ultimate tensile strength of the Trevira cord was reduced by approximately 12% after conditioning in saline (37°C) for 25 days. 

A comparison of the material properties between those previously described in the literature and in the presented study is not feasible because of the different test arrangements. The described tumor prosthesis is a hinged knee system and allows no rotational torque of the alloplastic cord. In contrast, previously tested cords were drilled when using them for reconstruction of the cruciate ligament. Furthermore, in our test arrangement the cord was twofold fixed in the metal block, whereas the previously tested ones were only fixed in a single layer technique. 

Our tests showed that the Trevira cord in combination with a metal block fixation device can temporarily achieve sufficient stability and extensor function in daily-life activity until such time as newly formed scar tissue can take over function. The results of our study suggest that the maximum material endurance of both the cord and the surface finish of the edge of the proximal fixation block is important. During the lifespan of the artificial cord providing active extension, biological tissue can form and eventually take over function of the extensor mechanism in the long term as in vivo and in vitro studies have already shown [39]. To avoid an extension lag, a high primary and midterm stability are essential, stressing the importance of pretensioning the artificial ligament at the time of implantation.

Clinical results, published by Plötz et al. [25], showed that the investigated fixation mechanism can bridge a crucial time gap. In 15 patients with malignant tumors of the proximal tibia, the patellar ligament of 5 patients was fixed with various techniques to the prostheses. In 10 of those 15 patients, the extensor mechanism was reconstructed with the 10 mm wide Trevira cord, fixed to a metal fixation block of the MML prosthetic system, similar to that experimentally tested (mean followup was 4.2 years). Mechanical failure occurred in two cases. Although the Trevira cord was ruptured in these cases, the extensor mechanism of these persons kept working well, because newly formed scar tissue took over function. 

In this experimental study, we found two screw failures when the original metal block with regular drill holes for the screws was used. In contrast, no screws failed when combined with the modified metal block with oblong holes giving the device an advantageous design with better fit. Given the fact that avoiding any bending and shear forces on the screws is most important for their durability, proper fit between the fixation block and the proximal edge of its notch is essential and can be easily achieved by oblong or bigger screw holes in the block. For this reason, the design of the MML tumor prosthetic knee system was changed according to our findings. 

The mean lag of active extension in the study of Plötz et al. [25] was 7.7° (0°–20°). In contrast, in a study of Petschnig et al. [57], in which the extensor apparatus was reconstructed by transposition of the fibula (mean followup 8 years), it was 12.5° (3°–15°). This group was compared to five patients (mean followup 5 years) having an extension deficit of 32.5° (18°–36°) after transposition of the M. gastrocnemius. Due to the superior functional results of the patients treated with Trevira cord reconstruction of the patellar ligament by Plötz et al., we recommend the use of this alloplastic material to achieve immediate postoperative stable fixation of the extensor mechanism. This gives the chance for exact adjustment and high pretension over a period of time long enough to allow scar tissue formation and strong enough to provide function without or with only minor extension lag. The use of an alloplastic material and the described fixation mechanism offers the possibility to combine it with other biological reconstructive procedures. 

##  Authors' Contributions

H. Rechl: Acquisition of data, analysis and interpretation of data, final approval of manuscript. B. M. Holzapfel: Drafting of the manuscript, analysis and interpretation of data. S. Lehner: Acquisition of data, analysis and interpretation of data, statistical analysis. H. Pilge: Analysis and interpretation of data, acquisition of data. H. Gollwitzer: Drafting of the manuscript, design, acquisition of data. E. Steinhauser: Conception and design, revision and final approval of the manuscript. 

## Figures and Tables

**Table 1:** Mean ultimate tensile strength and mean stiffness of the investigated fixation mechanism with Trevira cords. Tested as received and conditioned in saline (37°C) for 25 days.

	Ultimate tensile strength F_max_ (SD) (N)	Stiffness S_max_ (SD) (N/mm)
As received	2558 (±40)	136.5 (±4.9)
Conditioned	2248 (±88)	126.0 (±9.1)

**Table 2:** Strength and elongation of fixed Trevira cords.

	Mean value (SD)
Yield strength F_lin_ (N) (start of the plastic deformation)	768 (±42)
Ultimate tensile strength F_max_ (N)	2015 (±127)
Yield elongation d_lin_ (%)	5.8 (±0.7)
Ultimate elongation d_max_ (%)	31.3 (±6.0)
Stiffness S_max_ (N/mm) (normalized on a cord length of 50 mm)	343 (±50)

## References

[1] Goorin AM, Abelson HT, Frei E (1985). Osteosarcoma: fifteen years later. *New England Journal of Medicine*.

[2] Mirabello L, Troisi RJ, Savage SA (2009). Osteosarcoma incidence and survival rates from 1973 to 2004: data from the surveillance, epidemiology, and end results program. *Cancer*.

[3] Bacci G, Ferrari S, Lari S (2002). Osteosarcoma of the limb. Amputation or limb salvage in patients treated by neoadjuvant chemotherapy. *Journal of Bone and Joint Surgery. British*.

[4] Lindner NJ, Ramm O, Hillmann A (1999). Limb salvage and outcome of osteosarcoma: the university of Muenster experience. *Clinical Orthopaedics and Related Research*.

[5] Aho AJ, Ekfors T, Dean PB, Aro HT, Ahonen A, Nikkanen V (1994). Incorporation and clinical results of large allografts of the extremities and pelvis. *Clinical Orthopaedics and Related Research*.

[6] Matsuo T, Sugita T, Shimose S (2008). Rigid bridging of massive femur defect using double vascularized fibula graft with hydroxyapatite. *Archives of Orthopaedic and Trauma Surgery*.

[7] Otsuka T, Okuda T, Sekiya I, Tsuji H, Matsui N, Nishi G (1998). Rotation plasty for osteosarcoma of the femur. *Journal of Reconstructive Microsurgery*.

[8] Wirganowicz PZ, Eckardt JJ, Dorey FJ, Eilber FR, Kabo JM (1999). Etiology and results of tumor endoprosthesis revision surgery in 64 patients. *Clinical Orthopaedics and Related Research*.

[9] Gerdesmeyer L, Gollwitzer H, Diehl P, Burgkart R, Steinhauser E (2006). Reconstruction of the extensor mechanism in revision total knee arthroplasty and tumor surgery. *Orthopade*.

[10] Rechl H, Gradinger R, Ascherl R, Kaddick CH, Hipp E, Tan SK Patellar ligament reconstruction in tumors of the proximal tibia.

[11] Cool WP, Carter SR, Grimer RJ, Tillman RM, Walker PS (1997). Growth after extendible endoprosthetic replacement of the distal femur. *Journal of Bone and Joint Surgery. British*.

[12] Delepine G, Delepine N (1988). Preliminary results of 79 massive bone allografts in the conservative treatment of malignant tumors in adults and children. *International Orthopaedics*.

[13] Malawer MM, Price WM (1984). Gastrocnemius transposition flap in conjunction with limb-sparing surgery for primary bone sarcomas around the knee. *Plastic and Reconstructive Surgery*.

[14] Horowitz SM, Lane JM, Otis JC, Healey JH (1991). Prosthetic arthroplasty of the knee after resection of a sarcoma in the proximal end of the tibia: a report of sixteen cases. *Journal of Bone and Joint Surgery. Series A*.

[15] Anract P, Missenard G, Jeanrot C, Dubois V, Tomeno B (2001). Knee reconstruction with prosthesis and muscle flap after total arthrectomy. *Clinical Orthopaedics and Related Research*.

[16] Jaureguito JW, Dubois CM, Smith SR, Gottlieb LJ, Finn HA (1997). Medial gastrocnemius transposition flap for the treatment of disruption of the extensor mechanism after total knee arthroplasty. *Journal of Bone and Joint Surgery. Series A*.

[17] Osanai T, Tsuchiya T, Ogino T (2008). Gastrocnemius muscle flap including Achilles tendon after extensive patellectomy for soft tissue sarcoma. *Scandinavian Journal of Plastic and Reconstructive Surgery and Hand Surgery*.

[18] Bickels J, Wittig JC, Kollender Y (2001). Reconstruction of the extensor mechanism after proximal tibia endoprosthetic replacement. *Journal of Arthroplasty*.

[19] Jeon DG, Kim MS, Cho WH, Song WS, Lee SY (2007). Pasteurized autograft-prosthesis composite for distal femoral osteosarcoma. *Journal of Orthopaedic Science*.

[20] Barrack RL, Stanley T, Butler RA (2003). Treating extensor mechanism disruption after total knee arthroplasty. *Clinical Orthopaedics and Related Research*.

[21] Kulkarni S, Sawant M, Ireland J (1999). Allograft reconstruction of the extensor mechanism for progressive extensor lag after total knee arthroplasty and previous patellectomy. A 3-year follow-up. *Journal of Arthroplasty*.

[22] Leopold SS, Greidanus N, Paprosky WG, Berger RA, Rosenberg AG (1999). High rate of failure of allograft reconstruction of the extensor mechanism after total knee arthroplasty. *Journal of Bone and Joint Surgery. Series A*.

[23] Dominkus M, Sabeti M, Toma C, Abdolvahab F, Trieb K, Kotz RI (2006). Reconstructing the extensor apparatus with a new polyester ligament. *Clinical Orthopaedics and Related Research*.

[24] Gosheger G, Hillmann A, Lindner N (2001). Soft tissue reconstruction of megaprostheses using a trevira tube. *Clinical Orthopaedics and Related Research*.

[25] Plötz W, Rechl H, Burgkart R (2002). Limb salvage with tumor endoprostheses for malignant tumors of the knee. *Clinical Orthopaedics and Related Research*.

[26] Aracil J, Salom M, Aroca JE, Torro V, Lopez-Quiles D (1999). Extensor apparatus reconstruction with Leeds-Keio ligament in total knee arthroplasty. *Journal of Arthroplasty*.

[27] Fujikawa K, Ohtani T, Matsumoto H, Seedhom BB (1994). Reconstruction of the extensor apparatus of the knee with the Leeds-Keio ligament. *Journal of Bone and Joint Surgery. British*.

[28] Krudwig WK (2002). Anterior cruciate ligament reconstruction using an alloplastic ligament of polyethylene terephthalate (PET—Trevira—hochfest). Follow-up study. *Bio-Medical Materials and Engineering*.

[29] Mockwitz J (1985). Alloplastic reconstruction of the ligament of the knee in chronic rotation instabilities—technics and results. *Unfallchirurgie*.

[30] Gosheger G, Hardes J, Ahrens H, Gebert C, Winkelmann W (2005). Endoprosthetic replacement of the humerus combined with trapezius and latissimus dorsi transfer: a report of three patients. *Archives of Orthopaedic and Trauma Surgery*.

[31] Gosheger G, Gebert C, Ahrens H, Streitbuerger A, Winkelmann W, Hardes J (2006). Endoprosthetic reconstruction in 250 patients with sarcoma. *Clinical Orthopaedics and Related Research*.

[32] Matziolis G, Drahn T, Perka C (2003). Spontaneous patellar tendon rupture in a patient with Ehlers-Danlos syndrome: a case reportSpontane patellarsehnenruptur beim Ehlers-Danlos-syndrom. *Unfallchirurg*.

[33] Zieren J, Maecker F, Neuss H, Müller JM (2002). Trevira mesh: a promising new implant for the treatment of abdominal hernias. *Langenbeck’s Archives of Surgery*.

[34] Hagemeister N, de Guise JA (2003). In vitro evaluation of combined graft deformation in anterior cruciate ligament reconstructions. *Journal of Biomechanics*.

[35] Kdolsky R, Reihsner R, Schabus R, Beer RJ (1994). Measurement of stress-strain relationship and stress relaxation in various synthetic ligaments. *Knee Surgery, Sports Traumatology, Arthroscopy*.

[36] Kock HJ, Sturmer KM (1992). Biocompatibility and ingrowth of Trevira prostheses following replacement of the cruciate ligaments. *Medical and Biological Engineering and Computing*.

[37] Letsch R (1994). Comparative evaluation of different anchoring techniques for synthetic cruciate ligaments—a biomechanical and animal investigation. *Knee Surgery, Sports Traumatology, Arthroscopy*.

[38] Trieb K, Blahovec H, Brand G, Sabeti M, Dominkus M, Kotz R (2004). In vivo and in vitro cellular ingrowth into a new generation of artificial ligaments. *European Surgical Research*.

[39] Dominkus M, Sabeti M, Kotz R (2005). Functional tendon repair in orthopedic tumor surgery. *Orthopade*.

[40] Gerdesmeyer L, Töpfer A, Kircher J, Grundei H, Diehl P (2006). The modular MML revision system in knee revision and tumor arthroplasty. *Orthopade*.

[41] Salis-Soglio G, Ghanem M, Meinecke I, Ellenrieder M, Klinger HM, Kirchhoff C (2010). The modular endoprosthetic system Munich-Luebeck (MML): potential applications and results in the lower extremities. *Orthopade*.

[42] Hirokawa S (1993). Biomechanics of the knee joint: a critical review. *Critical Reviews in Biomedical Engineering*.

[43] Ellis MI, Seedhom BB, Wright V (1984). Forces in the knee joint whilst rising from a seated position. *Journal of Biomedical Engineering*.

[44] Hirokawa S (1991). Three-dimensional mathematical model analysis of the patellofemoral joint. *Journal of Biomechanics*.

[45] Ellis MI, Seedhom BB, Wright V, Dowson D (1980). An evaluation of the ratio between the tensions along the quadriceps tendon and the patellar ligament. *Engineering in Medicine*.

[46] Huberti HH, Hayes WC, Stone JL, Shybut GT (1984). Force ratios in the quadriceps tendon and ligamentum patellae. *Journal of Orthopaedic Research*.

[47] Van Eijden TMGJ, Kouwenhoven E, Verburg J, Weijs WA (1986). A mathematical model of the patellofemoral joint. *Journal of Biomechanics*.

[48] Leszko F, Sharma A, Komistek RD, Mahfouz MR, Cates HE, Scuderi GR (2010). Comparison of in vivo patellofemoral kinematics for subjects having high-flexion total knee arthroplasty implant with patients having normal knees. *Journal of Arthroplasty*.

[49] Sharma A, Leszko F, Komistek RD, Scuderi GR, Cates HE, Liu F (2008). In vivo patellofemoral forces in high flexion total knee arthroplasty. *Journal of Biomechanics*.

[50] Van Eijden TMGJ, Kouwenhoven E, Weijs WA (1987). Mechanics of the patellar articulation. Effects of patellar ligament length studied with a mathematical model. *Acta Orthopaedica Scandinavica*.

[51] Andriacchi TP, Galante JO, Fermier RW (1982). The influence of total knee-replacement design on walking and stair-climbing. *Journal of Bone and Joint Surgery. Series A*.

[52] Andriacchi TP, Hurwitz DE (1997). Gait biomechanics and total knee arthroplasty. *The American Journal of Knee Surgery*.

[53] Bischoff JE, Hertzler JS, Mason JJ (2009). Patellofemoral interactions in walking, stair ascent, and stair descent using a virtual patella model. *Journal of Biomechanics*.

[54] Morrison JB (1970). The mechanics of the knee joint in relation to normal walking. *Journal of Biomechanics*.

[55] Siebert HR, Rueger JM, Pannike A (1985). Experimental studies of ligament replacement. *Unfallchirurgie*.

[56] Contzen H (1985). Material technical prerequisites and biological principles for alloplastic replacement of the cruciate ligament. *Unfallchirurgie*.

[57] Petschnig R, Baron R, Kotz R, Ritschl P, Engel A (1995). Muscle function after endoprosthetic replacement of the proximal tibia. Different techniques for extensor reconstruction in 17 tumor patients. *Acta Orthopaedica Scandinavica*.

